# Comparisons between end-effector and exoskeleton rehabilitation robots regarding upper extremity function among chronic stroke patients with moderate-to-severe upper limb impairment

**DOI:** 10.1038/s41598-020-58630-2

**Published:** 2020-02-04

**Authors:** Stephanie Hyeyoung Lee, Gyulee Park, Duk Youn Cho, Ha Yeon Kim, Ji-Yeong Lee, Suyoung Kim, Si-Bog Park, Joon-Ho Shin

**Affiliations:** 1 Department of Rehabilitation Medicine, National Rehabilitation Center, Ministry of Health and Welfare, Seoul, Republic of Korea; 2 Department of Rehabilitation Medicine, Graduate School of Hanyang University, Seoul, Republic of Korea; 3 Translational Research Center for Rehabilitation Robots, National Rehabilitation Center, Ministry of Health and Welfare, Seoul, Republic of Korea; 4 Department of Law, Hanyang University, Seoul, Republic of Korea; 5 Department of Rehabilitation Medicine, Hanyang University College of Medicine, Seoul, Republic of Korea

**Keywords:** Stroke, Randomized controlled trials

## Abstract

End-effector (EE) and exoskeleton (Exo) robots have not been directly compared previously. The present study aimed to directly compare EE and Exo robots in chronic stroke patients with moderate-to-severe upper limb impairment. This single-blinded, randomised controlled trial included 38 patients with stroke who were admitted to the rehabilitation hospital. The patients were equally divided into EE and Exo groups. Baseline characteristics, including sex, age, stroke type, brain lesion side (left/right), stroke duration, Fugl–Meyer Assessment (FMA)–Upper Extremity score, and Wolf Motor Function Test (WMFT) score, were assessed. Additionally, impairment level (FMA, motor status score), activity (WMFT), and participation (stroke impact scale [SIS]) were evaluated. There were no significant differences in baseline characteristics between the groups. After the intervention, improvements were significantly better in the EE group with regard to activity and participation (WMFT–Functional ability rating scale, WMFT–Time, and SIS–Participation). There was no intervention-related adverse event. The EE robot intervention is better than the Exo robot intervention with regard to activity and participation among chronic stroke patients with moderate-to-severe upper limb impairment. Further research is needed to confirm this novel finding.

## Introduction

Upper extremity dysfunction is a common complication after stroke, and it has been reported to affect approximately 85% of stroke survivors in the early stage^1^ and 50% in the chronic stage^2^. Impaired upper extremity function limits performance of activities of daily living (ADLs) and decreases social participation^3^. Novel therapeutic techniques have been introduced to promote upper extremity function, and one such technique is robotic rehabilitation.

Rehabilitation robots are capable of reducing the burden on therapists by substituting human intervention and providing ideal therapies that fulfil the following main principles of stroke rehabilitation: repetition, high intensity, and task specificity^4^. Thus, robotic intervention has been highlighted as a promising therapy. A recent multicentre randomised controlled trial showed better improvements in FMA scores with robot-assisted training on comparing robot-assisted training with usual care, but showed no significant difference in scores on comparing robot-assisted training with enhanced upper limb therapy. These findings indicate that robot-assisted training can reduce the burden for therapists but is not a definite superior option^5^. Many systematic reviews and meta-analyses on rehabilitation robots have been published in the last two decades. In 2012, Norouzi-Gheidari *et al*. summarised 10 trials that compared robotic therapy with dose-matched conventional therapy and reported no significant differences in Fugl–Meyer Assessment (FMA) of the upper extremity and Functional Independence Measure scores between the therapies^6^. However, with an increase in the number of randomised controlled trials, a recent review involving 38 trials reported a significant difference in the FMA–Upper Extremity score between robotic therapy and conventional therapy, with a better score for robotic therapy^7^.

Many rehabilitation robots for the upper extremity have been released and are available for clinical use. These robots have shown positive clinical results. Thus, healthcare professionals and patients have multiple choices among many kinds of robots; however, there is limited evidence to guide their choices. Physicians tend to prescribe ‘robot intervention’ rather than specify a particular robot, unlike medication prescription, when selecting robotic rehabilitation. So far, different rehabilitation robots have been considered broadly as rehabilitation robots per se, despite some differences in effectiveness.

Rehabilitation robots are generally categorised into end-effector (EE) and exoskeleton (Exo) types according to their mechanical structures^8^. EE robots are connected to patients at one distal point, and their joints do not match with human joints. Force generated at the distal interface changes the positions of other joints simultaneously, making isolated movement of a single joint difficult^8,9^. Exo robots resemble human limbs as they are connected to patients at multiple points and their joint axes match with human joint axes. Training of specific muscles by controlling joint movements at calculated torques is possible^8,9^. Recent systematic reviews have performed indirect comparisons by subgroup analysis and have demonstrated contradictory results for EE and Exo robots. Veerbeek *et al*. reported significant favourable results with regard to FMA–Upper Extremity for EE robots but not for Exo robots^7^. On the other hand, Bertani *et al*. reported significant favourable results with regard to arm function for Exo robots but not for EE robots; however, the risk of bias should be considered owing to the smaller sample size of Exo robots when compared with that of EE robots^10^. Although these indirect comparisons are helpful, they are limited by the heterogeneity in clinical studies, including design, population, outcomes, and intervention protocols.

Many new robotic devices have been developed; however, there are no guidelines or standard requirements with regard to the most appropriate robot subtype, extent of degrees of freedom, and approach (functionality based or impairment based) for favourable outcomes. To our knowledge, no head-to-head clinical trial comparing different types of rehabilitation robots has been performed. Such a comparison may help in the decision making of healthcare professionals with regard to rehabilitation robots and may ultimately offer more optimal rehabilitation for patients. In particular, there is a great need for a direct comparison study to clarify effects according to the types of robots, as robots are expensive.

Therefore, we performed a randomised controlled trial to directly compare EE and Exo robots in a selected population of chronic stroke patients with moderate-to-severe upper limb impairment. The InMotion2 (Interactive Motion Technologies, Watertown, MA, USA) and Armeo Power (Hocoma, Volketswil, Switzerland) robots were selected as representative EE and Exo robots, respectively, among commercially available robots for their proven efficacy and safety, as well as accessibility around hospitals^11–14^.

## Methods

### Study design

This single-blinded, randomised controlled trial was conducted at a single rehabilitation hospital. Participants were randomly allocated to an EE group and Exo group (1:1 ratio) by using concealed envelopes with a card representing the group assignment. Occupational therapists who carried out assessments were blinded to group allocation. The study was approved by the Ethics Committee of the National Rehabilitation Center, Korea and was carried out in accordance with the approved guidelines. Written informed consent was provided by all participants. The study was registered at ClinicalTrials.gov (NCT03104881).

### Participants

For enrolment, the study considered 92 patients with stroke who were admitted to the rehabilitation hospital between March 2015 and August 2016. The inclusion criteria were as follows: (1) unilateral hemiplegic upper extremity dysfunction secondary to a unilateral ischaemic or haemorrhagic brain lesion; (2) stroke duration > 3 months; (3) FMA–Upper Extremity score of 8–30 for the affected upper extremity; and (4) ability to follow simple instructions. The exclusion criteria were as follows: (1) age < 20 years or > 80 years; (2) previous ischaemic or haemorrhagic stroke; (3) shoulder or elbow spasticity with a modified Ashworth scale (MAS) score ≥ 2; (4) severe upper extremity pain that could interfere with rehabilitation therapy; (5) neurological disorders other than stroke that can cause motor deficits, such as Parkinson’s disease, spinal cord injury, traumatic brain lesion, brain tumour, and peripheral neuropathy; and (4) uncontrolled severe medical conditions. Of the 92 patients, 53 did not meet the inclusion criteria or declined to participate. Thus, 39 patients were finally enrolled.

### Intervention

All participants received robot-assisted therapy with InMotion2 (EE group) or Armeo Power (Exo group) (30 minutes of active therapy 5 days a week for 4 weeks [total 20 sessions]) along with conventional occupational therapy (30 minutes of therapy [total 20 sessions]). Both robot-assisted therapies were managed by the same experienced research physical therapist. The therapy period was quantified by considering the active intervention time and not the time for preparations, such as attaching the robot to the patient and aligning the axis of the robot to that of the patient. Conventional occupational therapy involved range of motion exercises, strengthening exercises for the affected upper extremity, and basic ADL training. Overall, the same dosing parameters, including frequency and duration, were applied in the EE and Exo groups.

#### EE group

**Figure 1:**
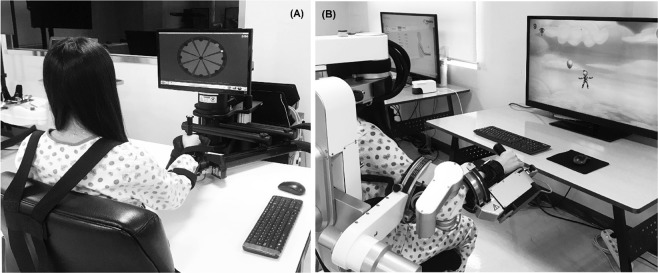
Two types of rehabilitation robots used for the robot-assisted therapy (**A**) InMotion2 for the EE group and (**B**) Armeo Power for the Exo group. EE, end-effector; Exo, exoskeleton.

The EE robot InMotion2 was used in the EE group. In the seated position, each participant held the handle attached to an arm support and performed goal-directed reaching movements in the gravity-compensated horizontal plane with two degrees of freedom, including the shoulder and elbow joints. From the starting point in the centre, the participant was instructed to move the handle toward eight targets positioned 45 degrees apart in circular arrangements, and the position of the handle was marked on the screen for real-time visual feedback (Fig. 1A). Reaching movements were supported through an assist-as-needed control system when targets could not be reached independently.

#### Exo group

The Exo robot Armeo Power was used in the Exo group. An exoskeleton with two adjustable cuffs at the upper and lower arms supported the arm weight with six degrees of freedom, including shoulder, elbow, and wrist joints, allowing extensive three-dimensional movements (Fig. 1B). An assist-as-needed control system assisted with the completion of tasks according to the participant’s capability. Each 30-minute session involved six training modules of 5 minutes each, which were tailored to each participant according to the severity of motor impairment and were mainly composed of goal-oriented movements that focused on the shoulder and elbow.

### Outcome measures

Baseline characteristics of the participants, including sex, age, stroke type, brain lesion side (left or right), stroke duration, FMA–Upper Extremity score, and Wolf Motor Function Test (WMFT) score, were assessed. We attempted to cover every domain of upper extremity functioning according to the International Classification of Functioning, Disability and Health (ICF), including impairment level (FMA, motor status score [MSS]), activity (WMFT), and participation (stroke impact scale [SIS])^15^.

All outcomes, except the SIS score, were assessed at baseline (T0), after 4 weeks of the intervention (T4), 4 weeks after the end of the intervention (T8), and 6 weeks after the end of the intervention (T10). The SIS score was assessed at T0 and T4.

#### Primary outcomes

The primary outcomes were changes in the FMA–Upper Extremity score (FMA–Total and FMA–Proximal [FMA–Prox]) and WMFT score (WMFT–Functional ability rating scale [FAS] and WMFT–Time), which reflect impairment and activity, respectively, at T4. FMA is an indicator of the level of impairment of the upper extremity, with higher scores indicating lower impairment^16^. We used the upper extremity section of the FMA (FMA–Total; score range from 0 to 66) and FMA–Prox (shoulder, elbow, and forearm; score range from 0 to 42) in the present study because the target of our robot-assisted therapy was the proximal portion of the upper extremity.

WMFT, as an activity indicator, has 15 items for testing functional ability and two items for testing the strength of the upper extremity^17^. Each of the 15 items for functional ability is graded with an ordinal scale from 0 to 5, and thus, the total WMFT–FAS score ranges from 0 to 75, with higher scores indicating better function. WMFT–Time refers to the total time in seconds needed to perform the designated 15 tasks, with a maximum of 120 seconds for each task. The log-transformed WMFT–Time score was used because the data had a skewed distribution. The strength of the upper extremity was quantified by WMFT–Weight, which is the sum of the following two items: forearm cuff weight and grip strength tested using a dynamometer.

#### Secondary outcomes

The secondary outcomes were changes in the FMA–Upper Extremity score (FMA–Total and FMA–Prox), WMFT score (WMFT–FAS, WMFT–Time, and WMFT–Weight), and MSS at T8 and T10, and changes in the SIS score at T4.

The MSS was used to evaluate the impairment level, as fine grading and voluntary movement specification are possible^18^. We used the total MSS (MSS–Total; score range from 0 to 82) and MSS–Shoulder and elbow section (MSS–Prox; score range from 0 to 40), with higher scores indicating lower impairment.

The SIS version 3.0 stroke-specific, self-reported questionnaire about health-related quality of life was used to evaluate participation. It includes the following eight domains: strength, hand function, ADLs and instrumental ADLs (ADLs/IADLs), mobility, communication, emotion, memory and thinking, and social participation. The score for each domain ranges from 0 to 100, with higher scores indicating a better health status^19^. Among the eight domains, SIS–Hand, SIS–Strength, SIS–ADLs/IADLs, and SIS–Social participation, which are thought to be related to upper extremity function, were selected for secondary outcome assessment. The overall SIS score (sum of all domain scores) was used to reflect the overall participation level. Additionally, the composite SIS score was used to demonstrate the comprehensive upper extremity function from the perspective of the ICF, with the sum of the SIS–Hand, SIS–ADLs/IADLs, and SIS–Social participation scores.

### Statistical analysis

Among the enrolled participants, those who completed 4 weeks of the intervention were included in the analyses. For missing T8 or T10 assessment, a last-observation-carried-forward method was used assuming no changes after the last observed status. To compare the baseline characteristics between the study groups, Student’s *t*-test or the Mann–Whitney *U* test was used for continuous variables depending on normality and the χ^2^ test was used for categorical variables. A repeated measures analysis of variance was performed with the Group (EE or Exo) as the between-group factor and Time (T0, T4, T8, or T10) as the within-group factor to compare the effects of each intervention on the outcome measures. The main effects of Time and the Time × Group interaction were evaluated at T4 to compare immediate effects in the EE and Exo groups. The same statistical methods were performed at T8 and T10 to determine how long the treatment effect was maintained. The Greenhouse–Geisser procedure was used when the assumption of sphericity was violated. All statistical analyses were performed using R 3.5.0 (http://www.r-project.org) and the R package psych for repeated measures analysis of variance^18^. A p-value < 0.05 was considered statistically significant.

## Results

**Figure 2:**
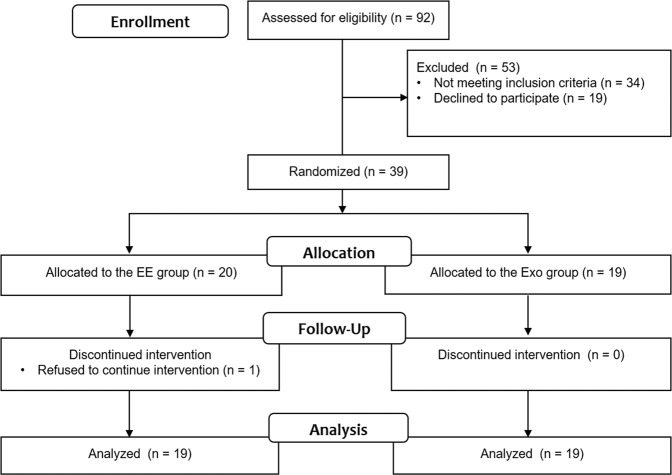
Study flowchart.

Thirty-nine participants were randomly allocated to the EE or Exo group. However, one participant who did not complete the intervention session was excluded from the analysis. Thus, 19 participants were finally present in each group (Fig. 2). There were no significant differences in baseline characteristics, including baseline primary outcome measures, between the EE and Exo groups (Table 1).

**Table 1:** Participant characteristics.

	EE (n = 19)	Exo (n = 19)	p-value
Age (years)	54.00 ± 10.01	49.47 ± 10.88	0.19^*^
Sex, male	11 (57.9)	15 (78.9)	0.16^†^
Time from stroke, months	6.42 ± 5.06	8.26 ± 7.57	0.56^‡^
Affected arm, right	9 (47.4)	11 (57.9)	0.52^†^
Stroke type, infarction	10 (52.6)	5 (26.3)	0.97^†^
FMA–Proximal	10.68 ± 3.97	10.68 ± 3.97	0.73^‡^
FMA–Total	15.37 ± 5.14	15.26 ± 4.37	0.75^‡^
WMFT–FAS	8.63 ± 6.92	9.89 ± 6.48	0.58^‡^
WMFT–Weight	1.74 ± 5.95	2.11 ± 2.77	0.091^‡^
WMFT–Time	3.09 ± 0.12	3.06 ± 0.13	0.34^‡^

Values are presented as mean ± standard deviation or number (%). ^*^t-test, ^†^χ^2^ test, ^‡^Mann–Whitney *U* test. EE, end-effector; Exo, exoskeleton; FMA, Fugl–Meyer Assessment; WMFT, Wolf Motor Function Test; FAS, functional ability rating scale.

### Primary outcome measures

**Figure 3:**
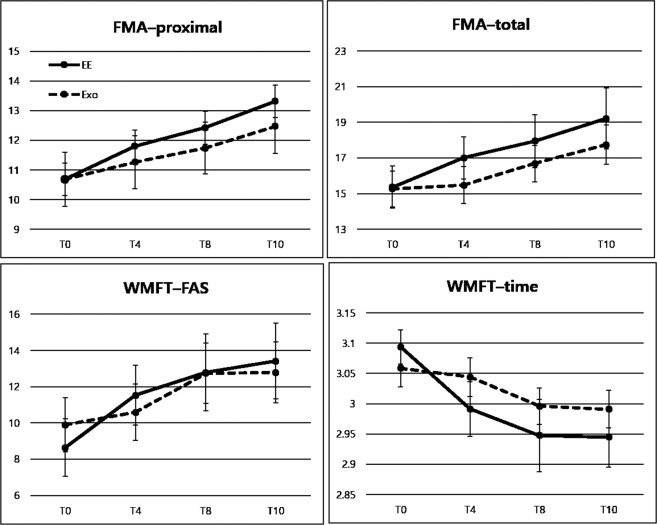
Changes of primary outcomes over time in the EE and Exo groups. Values are presented as mean ± standard error. EE, end-effector; Exo, exoskeleton.

The primary outcome measures are presented in Table 2 and Fig. 3. FMA–Total at T4 showed effects of Time (F = 3.123, df = 1, p = 0.086) and Time × Group (F = 1.859, df = 1, p = 0.181). Similarly, FMA–Prox at T4 showed effects of Time (F = 3.460, df = 1, p = 0.071) and Time × Group (F = 0.338, df = 1, p = 0.565).

**Table 2:** Comparisons of performance changes at T4, T8, and T10 in the EE and Exo groups.

	EE (n = 19)				Exo (n = 19)				Interaction	
	T0	T4	T8	T10	T0	T4	T8	T10	F	p-value
FMA–Total	15.4 ± 5.1	17.0 ± 5.2	17.9 ± 6.5	19.2 ± 7.4	15.3 ± 4.4	15.5 ± 4.5	16.7 ± 4.5	17.7 ± 4.8	0.809	0.492
FMA–Prox	10.7 ± 4.4	11.8 ± 4.1	12.4 ± 4.7	13.3 ± 5.3	10.7 ± 4.0	11.3 ± 4.0	11.7 ± 3.8	12.5 ± 4.0	0.410	0.746
WMFT–FAS	8.6 ± 6.9	11.5 ± 7.2	12.8 ± 9.3	13.4 ± 9.1	9.9 ± 6.5	10.6 ± 6.8	12.7 ± 7.3	12.8 ± 7.3	1.222	0.305
WMFT–Time	3.1 ± 0.1	3.0 ± 0.2	2.9 ± 0.3	2.9 ± 0.2	3.1 ± 0.1	3.0 ± 0.1	3.0 ± 0.1	3.0 ± 0.1	2.595	0.056
WMFT–Weights	1.7 ± 6.0	2.4 ± 6.8	2.8 ± 6.4	2.8 ± 7.1	2.1 ± 2.8	3.1 ± 3.4	3.4 ± 3.4	3.5 ± 3.9	0.141	0.936
MSS– Total	9.0 ± 5.1	11.1 ± 5.7	11.9 ± 6.0	13.2 ± 6.8	8.8 ± 4.5	10.0 ± 4.6	11.0 ± 5.5	11.0 ± 5.8	0.802	0.500
MSS–Prox	8.1 ± 4.2	9.3 ± 4.8	10.8 ± 5.0	12.0 ± 5.4	8.0 ± 3.5	9.1 ± 4.1	10.3 ± 4.4	10.4 ± 4.9	0.940	0.424
SIS–Hand	5.5 ± 10.7	20.5 ± 31.1			7.9 ± 19.4	14.2 ± 20.5			1.277	0.266
SIS–Strength	14.1 ± 13.3	17.4 ± 20.0			19.7 ± 19.5	21.4 ± 15.6			0.064	0.803
SIS–ADLs/IADLs	48.4 ± 22.8	50.0 ± 20.9			45.4 ± 21.2	48.4 ± 18.6			0.098	0.756
SIS–Social participation	49.7 ± 36.5	65.1 ± 33.2			65.9 ± 28.1	62.8 ± 32.6			3.270	0.079
SIS–Composite	103.7 ± 44.7	135.6 ± 66.7			119.3 ± 25.7	124.4 ± 40.6			2.539	0.120
SIS–Overall	382.2 ± 124.6	422.7 ± 141.7			371.0 ± 79.3	414.6 ± 81.9			0.0039	0.845

Values are presented as mean ± standard deviation. EE, end-effector; Exo, exoskeleton; FMA, Fugl–Meyer Assessment; WMFT, Wolf Motor Function Test; FAS, functional ability rating scale; MSS, motor status score; SIS, stroke impact scale; ADLs, activities of daily living; IADLs, instrumental activities of daily living.

Significant effects of Time × Group were noted at T4 for WMFT–FAS (F = 4.422, df = 1, p = 0.043) and WMFT–Time (F = 6.667, df = 1, p = 0.014). Additionally, significant effects of Time were noted at T4 for WMFT–FAS (F = 11.592, df = 1, p = 0.002) and WMFT–Time (F = 11.852, df = 1, p = 0.001). At T4, the mean changes of the WMFT–FAS score in the EE and Exo groups were 2.89 (standard error [SE]: 0.74) and 0.68 (SE: 0.74), respectively, and the mean changes of the log WMFT–Time score in the EE and Exo groups were 0.103 (SE: 0.024) and 0.015 (SE: 0.024), respectively.

### Secondary outcome measures

The secondary outcome measures at T4, T8, and T10 are presented in Table 2. Marginally significant effects of Time × Group were noted for log WMFT–Time at T8 (F = 2.995, df = 2, p = 0.056) and T10 (F = 2.595, df = 3, p = 0.056), and the mean decreases over the entire course of the study in the EE and Exo groups were 0.149 (SE: 0.026) and 0.067 (SE: 0.026), respectively. SIS–Social participation showed marginally significant effects of Time × Group (F = 3.270, df = 1, p = 0.079), and the mean changes in the EE and Exo groups at T4 were 3.14 (SE: 7.47) and −15.97 (SE: 7.47), respectively. Other outcomes did not show any Time × Group interaction. There was no adverse event related to the robot intervention.

## Discussion

This randomised controlled trial was performed to determine the most suitable robotic device for improving upper extremity function by directly comparing EE and Exo robots in patients with stroke. We found that improvements were significantly better in the EE group than in the Exo group with regard to activity and participation (WMFT–FAS, WMFT–Time, and SIS–Social participation) at the end of the intervention. Furthermore, the difference in WMFT–Time was somewhat maintained at follow-up with reduced significance.

There are several reasons for the better results in the EE group than in the Exo group after 4 weeks of the intervention. First, the intervention in the EE group was more impairment-based, while the intervention in the Exo group was more functional, and there was a fundamental difference between the two training approaches. Impairment-based training is a bottom-up approach with a focus on improving underlying capacity and is usually performed with simple repetitive movements, such as movements involved in the EE group. Functional training is a top-down approach with a focus on task performance of predefined goals and is usually performed with integrated movements, such as movements in game scenarios in the Exo group. Task-specific approaches are recommended for rehabilitation to promote recovery of body functions and activities^4^. Specific activities meaningful to the patient have been proven to produce cortical reorganisation and performance improvement, and reports have shown transfer of the effects of task-specific training to other untrained tasks^20,21^. Similarly, another study showed that functional occupational therapy was better than impairment-based occupational therapy when combined with robotic training^22^.

However, some contradictory results have raised concerns about the preference of functional training, especially for patients with moderate-to-severe impairment after stroke. A previous study showed that there was no significant difference between functional occupational therapy and robotic training^23^. Platz *et al*. demonstrated better results with systematic impairment-oriented training than with functional training in patients with severe impairment after stroke^24^. A previous pilot study compared simple planar reaching movements with reaching movements combined with grasp and release of an actual object and reported significantly better improvements in the FMA-Shoulder and Elbow subcomponents with impairment-based simple reaching training^25^. These results can be explained by the possibility that patients with moderate-to-severe impairment are not capable of performing different combinations of movements at the same time, and therefore, participation in functional training is very demanding. Thus, the bottom-up approach with a focus on one simple movement, which is referred to as an impairment-based approach, might be more beneficial in patients with moderate-to-severe impairment.

In addition, the superiority of impairment-based EE in the present study might be attributable to the combined effects of the two different strategies of impairment-based intervention and functional training. Previous studies, which adopted impairment-based training or functional training in addition to impairment-based robotic training, showed better effects with functional training than with impairment-based training^22,23^. On the other hand, we adopted robotic intervention adjunct to conventional occupational therapy, which involves both impairment-based intervention and functional training. Thus, the additive effects of robotic intervention might have been prominent in the high intensity, impairment-based EE intervention group in the present study.

Second, the difference in gravity support between the two robots might have a role. The EE robot trains patients in a two-dimensional horizontal plane with gravity compensation, whereas the Exo robot trains patients in a three-dimension area involving movements against gravity. The EE robot with full gravitational support decreases abnormal joint torque coupling of shoulder abduction and elbow flexion during movements and might reduce maladaptive compensatory movements during training^26,27^. Also on adding three-dimensional vertical movement robotic training to planar movement robotic training, no additional statistically significant improvements were observed. This might indicate that planar movement training with gravity compensation is an easier training approach that does not increase abnormal coupling of shoulder and elbow movements.

Third, the mechanical characteristics of the rehabilitation robots might have affected the results. Exo robots control multiple joints simultaneously, resulting in tight physical human-robot interaction, which may increase the burden on patients. Additionally, the high inertia of the Exo robot secondary to its complicated structure can interfere with manipulation. The EE robot has two degrees of freedom, whereas the Exo robot has six degrees of freedom. As our study participants were stroke patients with moderate-to-severe motor impairment, there is a possibility that the intervention with six degrees of freedom in a three-dimensional space was very challenging. The concept of ‘paradox of the diminishing number of degrees of freedom’ explains that to train a patient with severe motor impairment, the lowest number of degrees of freedom should be used first and the number of degrees of freedom should be gradually increased after improvements reach a plateau with the current number of degrees of freedom^28^. As fundamental impairment reduction occurs after learning to use the robot, including acquisition of novel sensorimotor interaction and visuomotor transformation, it might take more time to adapt to the Exo robot than the EE robot^29^.

Fourth, there was a difference in the number of repetitions between the EE and Exo groups. In order to reflect the true active components of the two interventions, we equalised the amount of time for active therapy rather than the time schedule for therapy, which includes time for preparations, such as wearing and removing the robot and aligning the axis of the robot to that of the participants^30^. Nonetheless, the number of repetitions was much lower in the Exo group than in the EE group, as the EE program involved highly repetitive simple impairment-based movements (planar reaching), whereas the Exo program involved comprehensive functional movements with high degrees of freedom, requiring a much longer time for each task. The practice amount is an important issue in rehabilitation, as a higher amount of rehabilitation is associated with greater improvement. Therefore, a future study comparing EE and Exo robots that adopts the same number of movements for a session might provide further insights into this issue. Nevertheless, our results favouring the EE robot are useful because improvement in efficiency or cost-effectiveness during the limited time allocated for therapy is important in the clinical setting.

The present study had some limitations. First, our findings were obtained for patients with moderate-to-severe impairment, and thus, they may not be similarly applicable to other patients. A previous crossover pilot study involving mildly impaired stroke patients failed to show a difference between multi-joint training and single-joint training using the same Exo robot^31^. Second, the intensity shortfall of our intervention might affect the results. Motor learning is more relevant to the Exo robot, which involves intrinsic or joint-based coordinates rather than extrinsic coordinates (EE robot)^32^. The process of motor learning needs sufficient training intensity; however, the extent of our intervention might not have been enough to induce motor learning. Thus, an increase in the therapy intensity might be needed in further studies. Third, differences between the groups were found for activity and participation, but not impairment. It was difficult to determine whether the significant changes resulted from restitution or compensation in our population, as impairment rarely changes 3 months after stroke, but compensation is possible 3 months after stroke^33,34^. Therefore, further studies involving patients with mild-to-moderate impairment who can manage higher degrees of freedom training or patients in the acute or subacute stroke phase are required to identify the optimal robot for each patient.

To our knowledge, this is the first clinical trial to directly compare EE and Exo rehabilitation robots. Moreover, to overcome the heterogeneity of the protocols in previous studies, therapies were performed for the same amount of active intervention time by the same experienced research physical therapist. Thus, our results represent the differential effects of the characteristics of the two rehabilitation robots, with minimisation of the confounding effects from the dose-response relationship. Overall, our study provides important information with regard to the clinical aspects of robot intervention, where data were limited to indirect comparisons with previous studies. This information may help guide decision-making in the clinical setting and may be useful for individualised interventions based on goals or patient characteristics.

## Conclusion

Our findings suggest that the EE robot intervention is better than the Exo robot intervention with regard to activity (WMFT–FAS and WMFT–Time) and participation (SIS–Participation) among chronic stroke patients with moderate-to-severe impairment of upper extremity function after 4 weeks of intervention. However, further studies are suggested to investigate the effects among patients with mild upper extremity impairment in order to confirm our explanation of suitability of impairment-based training for moderate-to-severe impairment and functional training for mild impairment. Additionally, studies comparing EE and Exo robots with movement number-matched sessions might provide further information.
